# Genetic Evaluation of Prelingual Hearing Impairment: Recommendations of an European Network for Genetic Hearing Impairment

**DOI:** 10.3390/audiolres13030029

**Published:** 2023-05-10

**Authors:** Laurence Jonard, Davide Brotto, Miguel A. Moreno-Pelayo, Ignacio del Castillo, Hannie Kremer, Ronald Pennings, Helena Caria, Graça Fialho, An Boudewyns, Guy Van Camp, Monika Ołdak, Dominika Oziębło, Naïma Deggouj, Romolo Daniele De Siati, Paolo Gasparini, Giorgia Girotto, Margriet Verstreken, Silvia Dossena, Sebastian Roesch, Saba Battelino, Katarina Trebušak Podkrajšek, Athanasia Warnecke, Thomas Lenarz, Anke Lesinski-Schiedat, Michel Mondain, Anne-Françoise Roux, Françoise Denoyelle, Natalie Loundon, Margaux Serey Gaut, Patrizia Trevisi, Elisa Rubinato, Alessandro Martini, Sandrine Marlin

**Affiliations:** 1 Centre de Référence «Surdités Génétiques», Fédération de Génétique, Centre de Recherche en Audiologie (CREA), Hôpital Necker-Enfants Malades, Assistance Publique Hôpitaux de Paris, 75015 Paris, France; 2 ENT Unit, Neurosciences Department, University of Padova, 35122 Padova, Italy; 3 Centro de Investigación Biomédica en Red de Enfermedades Raras (CIBERER), Instituto Ramón y Cajal deInvestigaciones Sani-tarias (IRYCIS), Genetics Department, University hospital Ramón y Cajal, 28034 Madrid, Spain; 4 Department of Otorhinolaryngology and Department of Human Genetics, Hearing & Genes, Radboud University Medical Center, Donders Institute for Brain, Cognition and Behaviour, Radboud University, 6525 XZ Nijmegen, The Netherlands; 5 Donders Institute for Brain, Cognition and Behaviour, Radboud University, 6525 XZ Nijmegen, The Netherlands; 6 BioISI—Biosystems & Integrative Sciences Institute, Faculty of Sciences, University of Lisboa, 1649-004 Lisboa, Portugal; 7 Biomedical Sciences Department, CIIAS—School of Health, Polytechnic Institute of Setubal, 2914-503 Setubal, Portugal; 8 Department of Otorhinolaryngology, Head and Neck Surgery, Antwerp University Hospital, University of Antwerp, 2000 Edegem, Belgium; 9 Center for Medical Genetics, University of Antwerp, 2000 Antwerp, Belgium; 10 Department of Genetics, Institute of Physiology and Pathology of Hearing, 02-042 Warsaw, Poland; 11 ENT Department, UCLouvain, Academic Hospital Saint-Luc-Brussels, 1200 Bruxelles, Belgium; 12 Medical Genetics, Institute for Ma-ternal and Child Health (IRCCS) “Burlo Garofolo”, Department of Medical, Surgery and Health Sciences, University of Trieste, 34127 Trieste, Italy; 13 European Institute for ORL, GZA Campus St Augustinus, 2610 Antwerp, Belgium; 14 Institute of Pharmacology and Toxicology, Paracelsus Medical University, 5020 Salzburg, Austria; 15 Department of Otorhinolaryngology, Head and Neck Surgery, Paracelsus Medical University, 5020 Salzburg, Austria; 16 Department of Otorhinolaryngology and Cervicofacial Surgery, University Medical Centre Ljubljana, Medical Faculty, University of Ljubljana, 1000 Ljubljana, Slovenia; 17 Institute of Biochemistry and Molecular Genetics, Medical Faculty, University of Ljubljana, 1000 Ljubljana, Slovenia; 18 Department of Otorhinolaryngology—Head and Neck Surgery, Hannover Medical School, D-30625 Hannover, Germany; 19 Cluster of Excellence Hearing4all, German Research Foundation, Oldenburg 26129, Germany; 20 Medical Head German Hearing Center, Department of Otorhinolaryngology, Medical University of Hannover, D-30625 Hannover, Germany; 21 ENT Department, CHU Montpellier, Université de Montpellier, 34090 Montpellier, France; 22 Laboratoire de Génétique Moléculaire, CHU de Montpellier, Université de Montpellier, 34090 Montpellier, France; 23 Service d’ORL Pédiatrique et de Chirurgie Cervico-Faciale, INSERM UMR 1120, Hôpital Necker-Enfants Malades, Assistance Publique Hôpitaux de Paris, 75015 Paris, France; 24 Laboratory of Embryology and Genetics of Malformations, Imagine Institute, INSERM UMR 1163, Université de Paris, 75015 Paris, France

## 1. Introduction

The cause of childhood hearing impairment (excluding infectious pathology of the middle ear) can be extrinsic (embryofoetopathy, meningitis, trauma, drug ototoxicity, noise trauma, etc.), genetic or both. In industrialized countries, the proportion of genetic sensorineural hearing impairment is currently estimated at 66%. Hereditary hearing loss can be classified into two broad categories: non-syndromic estimated at 70–90% and syndromic at 10–30%. Several hundred of syndromic associations have been described in which the hearing deficit may be associated with abnormalities or malformations in other organs [1]. Most inherited hearing impairments are monogenic but there is great genetic heterogeneity.

The knowledge of non-syndromic hearing loss has tremendously increased since 1995 and more than 120 genes have been described [2].

The development of routine molecular diagnosis has allowed the identification of a genetic cause in a large part of sporadic hearing losses previously classified as “unknown cause” and a genetic cause is now estimated to account for about 80% of causes of non-syndromic congenital sensorineural hearing impairment (NSCSHI). In practice, clinical genetics bring together two main activities: etiological diagnosis and genetic counseling performed in specialized hospital departments. Since 2004, centers aiming at the diagnosis, management, training and research in the field of rare diseases (reference centers for rare diseases) have been built in several European countries. Each genetic deafness network brings together clinical and molecular geneticists, ENTs and research teams. Using the same model and in parallel with the labeling of the ERN (European rare network), a European network for genetic deafness was created in 2017.

## 2. Families’ Expectations Regarding Genetic Investigations

The expectations of families regarding genetics are different and depend on the history of each family. However, “making sense” seems to be a common need for patients and their families. Indeed, the narcissistic injury caused by the passage from the dreamed child to the real child causes high expectations of understanding from parents. In the search for etiology, parents seek relief for feelings of guilt and anxiety. Therefore, during the genetic consultation, preconceived ideas, sometimes rooted for a long time in the family’s imagination, resurface. In addition to researching etiology, this consultation brings scientific and medical information on hearing loss. Obtaining a molecular result can also make the existence of a hearing defect concrete and “scientific”, thus offering relief by putting an end to a psychic blur.

To date, the goals of genetic evaluation of a CSHI are multiple:

- to establish the etiology of the hearing impairment;
- to identify associated features;
- to establish a prognosis of the hearing impairment;
- to improve the management of hearing loss;
- to evaluate the recurrence risk in parents’ and patient’s offspring (genetic counseling);
- to detect possibly other affected subjects within the family.

In the last decade, scientific and technological improvement as well as clinical experience has allowed us to change our practices. During genetic consultations, a thorough clinical examination, revision of the clinical history, a precise and complete interrogation on the personal (extrinsic factors, associated signs) and familial (ethnicity, possible consanguinity, hearing disorders, and possible associated signs) history of each patient are conducted. The phenotype of the hearing impairment is defined as: age of onset or at first diagnosis, evolution (stable, progressive or fluctuating), laterality/symmetry, degree (mild, moderate, severe or profound), type of deficit (sensorineural, conductive, mixed, auditory neuropathy), and shape of the audiometric curve are collected. In addition, attention should be paid to the acquisition of motor milestones, speech–language development and the presence of associated features such as tinnitus or vestibular symptoms. The results of complementary testing such as vestibular examination, and temporal bones, olfactory bulbs and brain imaging should be reviewed.

## 3. Mandatory Investigations for All Cases

The following examinations are systematically required:

- CMV serology in the child and mother (in order to exclude CMV fetopathy if at least one of the two is negative) and if possible CMV research on dried blood spot, urine or saliva samples (depending on the age of the child), collected as early as possible to diagnose prenatal CMV infection [3];
- Ophthalmological examination in order to take care of refractive problems for optimal visual acuity and to research visual pathologies associated within a syndromic form;
- Audiograms of the siblings to diagnose familial cases of variable severity or progressive hearing loss and other family members if indicated by pedigree analysis.

## 4. Other Investigations Required after Clinical Suspicion of Specific Syndromic Forms

Further examinations and investigations will be requested depending on the clinical context or on the results of comprehensive genetic testing:

- An electroretinogram in the case of a bilateral profound congenital hearing impairment with motor delay without malformation of the inner ear (suspicion of Usher syndrome type 1) [4];
- A renal ultrasound in the case of sensorineural and/or conductive hearing loss associated with preauricular tags or pits (suspicion of branchiootorenal syndrome (BOR)) [5];
- A dosage of free thyroid hormones, TSH and Tg, in the case of a hearing defect with bilateral dilatation of the vestibular aqueduct (suspicion of Pendred syndrome) [6];
- An electrocardiogram in the case of profound congenital bilateral deafness without inner ear malformations and without *GJB2* pathogenic variations (cf infra) (suspicion of Jervel–Lange-Nielsen syndrome);
- A specialized ophthalmological examination in case of an auditory neuropathy (search of an optical atrophy in a syndromic form);
- Audiograms of the parents at the slightest doubt of a hearing defect or in a second intention (undiagnosed cases).

## 5. Genetic Investigations

At the end of this consultation, molecular tests may be proposed if a genetic etiology is suspected.

For non-syndromic hearing loss, targeted molecular analysis are still proposed in some departments:

- The search for alterations at the DFNB1 locus (GJB2 pathogenic variations and/or GJB6 deletions: del(GJB6-D13S1830) and del(GJB6-D13S1854)): responsible for 20 to 30% of bilateral NSCSHIs. The imaging of the inner ear is normal in the vast majority of cases and there is no vestibular deficit [7,8,9]. The hearing loss is globally symmetrical ranging from mild to profound. If the deficit is not immediately profound, it can progress over time in about 20% of cases;
- The search for STRC pathogenic variations or alterations at the DFN16 locus: the phenotype is the same as the previous except that the hearing loss is mild or moderate without evolution over time [10,11];
- The search for SLC26A4 pathogenic variations: hearing impairment is often prelingual, immediately or rapidly bilateral, sometimes asymmetrical, fluctuating and often progressive [6]. Temporal bone imaging systematically finds a bilateral dilatation of the vestibular aqueducts (CT scan) and endolymphatic ducts (MRI); frequently associated with incomplete cochlear partition type 2 malformation (Mondini malformation) [12];
- The search for OTOF pathogenic variations: this gene remains the most frequently involved in isolated early auditory neuropathy/synaptopathy [13,14];
- The search for POU3F4 pathogenic variations or alterations at the DFNX2 locus: moderate to profound bilateral congenital isolated, mixed or sensorineural hearing loss among males and moderate postlingual, mixed or sensorineural hearing loss among females [15]. Among males and some females, temporal bone imaging also identifies a bilateral important malformation of the auditory canals and cochlea (incomplete partition type 3) [16].

All these genes are responsible for bilateral NSCSHI. The genetic causes of unilateral non-syndromic hearing loss have not been elucidated to date.

The great genetic heterogeneity of prelingual hearing impairments makes it very interesting to use a recent high-throughput sequencing (NGS: next generation sequencing) technology to simultaneously analyze a large number of genes [17,18]. The technological power of this method makes it possible to establish a genetic diagnosis in a large number of cases. This type of molecular testing is offered by several specialized genetic services in Europe; each laboratory being free to choose the deafness genes to analyze by NGS.

For syndromic hearing loss, we can distinguish two frequently encountered situations:

- The clinical presentation suggests a known syndromic diagnosis (for example: Waardenburg, Usher, Pendred, BOR syndromes, etc.), and the clinician will ask for an analysis of the gene(s) known to be responsible for this phenotype (targeted analysis or NGS panel testing) [19];
- The patient has a polymalformative syndrome or a set of clinical signs associated with deafness that does not evoke a known diagnosis, and the clinician will most often request a chromosome assessment by a CGH array (comparative genomic hybridization array). This technique makes it possible to identify an abnormal number of copies of a chromosomal segment (deletion or duplication = copy number variation). These chromosomal abnormalities may involve one to hundreds of different genes located contiguously on a chromosome segment. An unusual association of clinical signs may be due to a fortuitous association of several genetic defects.

Regardless of the technology used, the confirmation of a molecular genetic result also requires the analysis of all deaf siblings and both parents. Indeed, it is essential to verify that the family segregation of the genetic pathogenic variants identified in the patient matches with the inheritance of the hearing loss. In light of this, it could also be necessary to carry out genetic and phenotypic analyses of available healthy members of the family in order to better characterize the “Variants of Uncertain Significance” which still remain a huge difficulty for laboratories, clinicians, and patients alike [20].

Finally, even if the sequence of all our genes (nearly 20.000 per individual) is known, many of these are not yet related to a human pathology. For about 30% of congenital hearing impairments the analysis of known genes does not identify the causative anomaly. The implication of new genes in hearing defects currently involves the analysis (sequencing) of all the genes (exome) of one or more patients. This technology remains mostly in the field of research. However, the identification of new genetic causes of hearing loss would make it possible to increase the diagnostic rate [21].

Regarding hearing impairment, genetic evaluation is rarely motivated by a request for genetic counseling in terms of prenatal diagnosis or preimplantation diagnosis. Most prenatal diagnosis applications for hearing defects are for syndromic forms in which hearing loss is associated with other disabilities or malformations, for example: Treacher–Collins syndrome, mitochondrial diseases, Alport syndrome, Usher syndrome or BOR syndrome. Couples asking for prenatal diagnosis in case of non-syndromic hearing loss are rare. Regardless of the context, both the availability of molecular tests and the legislative framework of each country must be considered.

To date, no curative treatment is available in cases of sensorineural hearing impairment, but the identification of a genetic origin helps to better understand the normal functioning of the ear and the mechanisms at the origin of hearing impairment. This could be the first step towards the development of therapies, based on the determination of the causal genetic anomaly. In fact, the first studies of gene and/or cell therapies conducted in animals show promising prospects [22,23,24,25]. The use of these treatments in humans will go through the preliminary determination of the cause of hearing loss in each patient and the establishment of genotype/phenotype correlations over large cohorts, leading us to collaborate in international networks and sharing our data.

**Mandatory examinations for all cases**

1. Phenotype of proband case’s hearing impairment: age of onset, type of deficit, degree, evolution, laterality/symmetry and shape of the audiometric curve;
2. Complete clinical examination, personal and familial history;
3. CMV research on dried blood spot or CMV serology in the patient and their mother;
4. Ophthalmological examination;
5. Temporal bone and brain imaging;
6. Audiograms of the kinship.

**Principal genetic investigations**

1. Targeted molecular analysis: e.g., DFNB1 locus (GJB2 pathogenic variations and/or GJB6 deletions; symmetrical bilateral mild to profound NSCSHI with normal inner ear morphology and no vestibular deficit); DFN16 locus (STRC pathogenic variations or deletions, stable bilateral mild or moderate NSCSHI); SLC26A4 (prelingual fluctuating or progressive NSCSHI with bilateral dilatation of the vestibular aqueducts; OTOF (early bilateral auditory neuropathy/synaptopathy);
2. Chromosomal analysis by CGH array for polymalformative syndrome or a set of clinical signs associated with deafness that does not evoke a known diagnosis;
3. Next generation sequencing: High-throughput sequencing technology to simultaneously analyze a set of genes implicated in syndromic or non-syndromic HL;
4. Exome or genome analysis.
